# Impact of diastolic dysfunction severity on global left ventricular volumetric filling - assessment by automated segmentation of routine cine cardiovascular magnetic resonance

**DOI:** 10.1186/1532-429X-12-46

**Published:** 2010-07-31

**Authors:** Dorinna D Mendoza, Noel CF Codella, Yi Wang, Martin R Prince, Sonia Sethi, Shant J Manoushagian, Keigo Kawaji, James K Min, Troy M LaBounty, Richard B Devereux, Jonathan W Weinsaft

**Affiliations:** 1 Division of Cardiology, Department of Medicine, Weill Cornell Medical College, NY, NY, USA; 2 Department of Radiology, Weill Cornell Medical College, NY, NY, USA

## Abstract

**Objectives:**

To examine relationships between severity of echocardiography (echo) -evidenced diastolic dysfunction (DD) and volumetric filling by automated processing of routine cine cardiovascular magnetic resonance (CMR).

**Background:**

Cine-CMR provides high-resolution assessment of left ventricular (LV) chamber volumes. Automated segmentation (LV-METRIC) yields LV filling curves by segmenting all short-axis images across all temporal phases. This study used cine-CMR to assess filling changes that occur with progressive DD.

**Methods:**

115 post-MI patients underwent CMR and echo within 1 day. LV-METRIC yielded multiple diastolic indices - E:A ratio, peak filling rate (PFR), time to peak filling rate (TPFR), and diastolic volume recovery (DVR_80 _- proportion of diastole required to recover 80% stroke volume). Echo was the reference for DD.

**Results:**

LV-METRIC successfully generated LV filling curves in all patients. CMR indices were reproducible (≤ 1% inter-reader differences) and required minimal processing time (175 ± 34 images/exam, 2:09 ± 0:51 minutes). CMR E:A ratio decreased with grade 1 and increased with grades 2-3 DD. Diastolic filling intervals, measured by DVR_80 _or TPFR, prolonged with grade 1 and shortened with grade 3 DD, paralleling echo deceleration time (p < 0.001). PFR by CMR increased with DD grade, similar to E/e' (p < 0.001). Prolonged DVR_80 _identified 71% of patients with echo-evidenced grade 1 but no patients with grade 3 DD, and stroke-volume adjusted PFR identified 67% with grade 3 but none with grade 1 DD (matched specificity = 83%). The combination of DVR_80 _and PFR identified 53% of patients with grade 2 DD. Prolonged DVR_80 _was associated with grade 1 (OR 2.79, CI 1.65-4.05, p = 0.001) with a similar trend for grade 2 (OR 1.35, CI 0.98-1.74, p = 0.06), whereas high PFR was associated with grade 3 (OR 1.14, CI 1.02-1.25, p = 0.02) DD.

**Conclusions:**

Automated cine-CMR segmentation can discern LV filling changes that occur with increasing severity of echo-evidenced DD. Impaired relaxation is associated with prolonged filling intervals whereas restrictive filling is characterized by increased filling rates.

## Background

Left ventricular (LV) diastolic dysfunction (DD) has important consequences following acute myocardial infarction (MI) as heart failure and mortality risks are linked to severity of diastolic impairment [1-3]. DD alters timing and profiles of LV filling, which initially compensate for impaired LV relaxation but ultimately impede LV performance. Echocardiography (echo) identifies DD based on mitral inflow or myocardial compliance sampled at regional myocardial locations [4,5]. This approach is potentially limited by localized changes in LV contractility and myocardial tissue composition, affecting regional compliance but not necessarily impacting global diastolic performance [4,6]. Global LV filling curves, which have been previously employed using radionuclide imaging techniques such as RNCA and SPECT, provide an alternative means of assessing diastolic physiology based on timing and pattern of dynamic changes in LV chamber volumes [7-10].

Cardiovascular magnetic resonance (CMR) is a standard for LV chamber volumes and ejection fraction (EF) based on quantification of cine images acquired at end-diastole and end-systole [11,12]. Cine-CMR acquires dynamic images throughout the cardiac cycle and thereby contains intrinsic data concerning volumetric changes during diastole. Manual segmentation of cine-CMR is an impractical means of assessing LV filling as quantification of all slice positions across all temporal phases typically requires planimetry of over 150 images. We have recently developed an automated algorithm (LV-METRIC) that quantifies chamber volumes using routine cine-CMR. In initial validation studies, LV-METRIC agreed with manual planimetry of phantom and clinical volumes [13,14]. In another study, LV-METRIC filling curves by LV-METRIC differentiated between binary presence or absence of echo-evidenced DD [15]. However, all patients had normal systolic function, retrospective data was used for validation, and the relation between cine-CMR filling parameters and graded severity of DD was not evaluated.

The current study employed LV-METRIC among a broad post-MI population with variable systolic function. In all patients, dedicated echo imaging was prospectively done within one day of CMR to provide a uniform reference for diastolic performance. The aim of this study was to test whether automated segmentation of routine cine-CMR can discern LV filling changes that occur with graded severity of diastolic impairment.

## Methods

### Population

The population comprised consecutive patients enrolled in a post-MI imaging registry (clinical trial #NCT00539045) who underwent CMR and echo within one day. Patients were excluded if echo characterization of diastolic performance was incomplete due to absence of tissue Doppler or pulmonary vein inflow patterns (n = 6), arrhythmia prohibiting echo/CMR assessment of LV filling (n = 2), or intolerance of CMR (n = 1). No patients were excluded based on clinical characteristics or CMR processing results.

To minimize the impact of post-MI stunning and transient volume shifts on LV performance [16], imaging was performed during a pre-specified time frame of 20-40 days post-MI. Imaging was completed between August 2005 and December 2009 at Weill Cornell Medical College (WMC). The study was conducted in accordance with the WMC Institutional Review Board; all patients provided written informed consent.

### Imaging Protocol

#### Echocardiography

Transthoracic echoes were performed using commercial equipment (General Electric Vivid-7 or Siemens Sequoia). Echo measurements, adjudication of diastolic filling patterns, and classifications of diastolic grade were made by an experienced echocardiographer (RBD) blinded to CMR results. Mitral inflow parameters and tissue Doppler profiles were acquired in an apical 4-chamber view. Linear measurements of chamber size and systolic function were performed in accordance with consensus guidelines [17]. DD was classified based on both mitral inflow and tissue Doppler parameters [4]. Deceleration time cutoffs were in accordance with previously reported criteria and established standards in the WMC echo laboratory [1,15,18,19]. Diastolic performance was graded as follows:

• ***Normal: ***E/A ≥ 0.8, septal e' ≥ 8 cm/s, lateral e' ≥ 10 cm/s, deceleration time (DT) 140-240 msec

• ***Grade 1 (mild)***: E/A < 0.8, septal e' < 8 cm/s, lateral e' < 10 cm/s, DT > 240 msec

• ***Grade 2 (moderate)*: **E/A 0.8-1.5, septal e' < 8 cm/s, lateral e' < 10 cm/s, DT 140-240 msec

• ***Grade 3 (severe)*: **E/A ≥ 2, septal e' < 8 cm/s, lateral e' < 10 cm/s, DT < 140 msec

In patients with equivocal tissue Doppler indices (i.e. abnormal lateral, normal septal e' amplitude), e'/a' reversal (< 1) and pulmonary vein profiles were used to establish presence of DD [4,5].

#### CMR

CMR was performed using 1.5 Tesla scanners (General Electric). Cine-CMR used a commercially available 2D steady state free precession pulse sequence. Images were acquired in contiguous short-axis slices from the mitral annulus through the LV apex. Typical parameters were repetition time 3.5 msec, echo time 1.6 msec, flip angle 60°, in-plane spatial resolution 1.9 mm × 1.4 mm, slice thickness 6 mm, inter-slice gap 4 mm, reconstructed temporal resolution 36.5 ± 9.2 msec. Delayed enhancement CMR (DE-CMR) was performed in all patients for assessment of LV infarct size.

### Automated CMR Segmentation

#### LV Systolic Function and Morphology

Automated quantification of LV volumes and myocardial mass was performed using LV-METRIC. User input included identification of the slice range to be segmented and definition of the mitral and aortic valve annulus. Optional corrections comprised manual contouring to restrict region-growth and adjusting blood sensitivity. LV volumes were quantified as the sum of short axis chamber volumes (2D area * slice thickness) measured during end-diastole (EDV) and end-systole (ESV). Ejection fraction (EF) was calculated as EDV-ESV]/EDV*100. Automated border detection of end-diastolic endocardial and epicardial contours quantified LV mass, calculated as the product of myocardial volume and specific gravity ([EpiEDV-EDV]*1.05) [20].

#### Adjunctive Analyses

CMR images were analyzed to quantify other structural indices that can impact LV diastolic performance. Left atrial volume was quantified in accordance with guidelines for biplane chamber planimetry [17]. LV wall motion and infarct size were scored using a 17-segment model. Segmental infarction was based on transmural extent of hyperenhanced (bright) myocardium on DE-CMR and graded using a 5-point scale (0 = no hyperenhancement; 1 = 1-25%; 2 = 26-50%; 3 = 51-75%; 4 = 76-100%). Global infarct size as a percentage of LV myocardium was calculated by summing the segmental scores (each weighted by the midpoint of the range of hyperenhancement) and dividing by the total number of regions [21].

#### LV Diastolic Function

**Figure 1:**
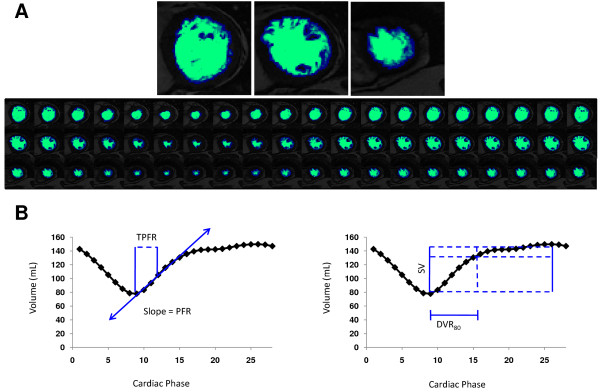
**Typical Example of Automated Segmentation**. **(A) **LV-METRIC segmentation of representative images from basal, mid, and apical slice locations (top). Segmentation was performed across spatial (vertical) and temporal (horizontal) domains for volumetric assessment of global LV filling (bottom). **(B) **Diastolic filling indices generated by LV-METRIC. Peak filling rate (PFR), defined as maximal slope of Δ volume/Δ temporal phase, and time to peak filling rate (TPFR) are shown on the left-sided graph. Diastolic volume recovery (DVR_80_), calculated as proportion of diastole necessary to recover a threshold of 80% LV stroke volume (SV), is shown on the right-sided graph.

LV-METRIC segmentation was performed for all LV short-axis slices across all temporal phases (Figure 1A) to assess the time course of global volumetric filling (Figure 1B). Volumetric data were low pass filtered to remove any non-physiologic high frequency components from the volume-filling curve. The purpose of the filter was to suppress small segmentation errors in the volume curve from being amplified in the derivative curve. The following diastolic parameters were evaluated:

• *Peak Filling Rate *[PFR] - maximal LV filling rate defined by maximal change in LV volume between sequential temporal phases (Δ volume/Δ phase). This index was also normalized for LV stroke volume [nPFR]

• *Time to Peak Filling Rate *[TPFR] - time interval between end-systole and peak filling rate

• *Diastolic 80% Volume Recovery *[DVR_80_] - proportion of diastole required to recover 80% LV stroke volume, a cutoff based on prior validation data [15]

**Figure 2:**
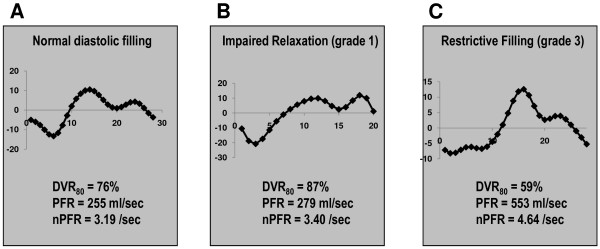
**Representative Filling Profiles**. Representative E:A profiles generated by cine-CMR for patients with normal echo-evidenced filling **(A)**, grade 1 **(B) **and grade 3 **(C) **DD. Note E:A reversal with prolonged DVR_80 _for grade 1, and augmented E:A ratio with increased PFR for grade 3 DD. The x-axis represents phase and the y-axis is volume per phase.

Figures 1B provides representative illustrations of each parameter. In addition, the first derivative of the volume-time curve was used to generate early (E) and late (A) filling profiles, similar to a typical echo mitral inflow pattern (Figure 2).

### Statistical Methods

Comparisons between groups were made using analysis of variance (for multiple group comparisons) or Student's t-test for continuous variables, with results expressed as mean ± standard deviation. Categorical variables were compared using the Chi-square test. Bivariate correlation was employed to evaluate associations between continuous echo and CMR parameters. Multinomial logistic regression was used to assess relations of CMR parameters to echo DD grade. Two-sided p < 0.05 was considered indicative of statistical significance. Statistical calculations were performed using SPSS 12.0 (SPSS Inc, Chicago, IL).

## Results

### Population Characteristics

The population consisted of 115 post-MI patients who underwent echo and CMR within a one day interval (97% same day); 65% had echo-evidenced DD. Diastolic dysfunction was mild (grade 1) in 28% (n = 21), moderate (grade 2) in 60% (n = 45), and severe (grade 3) in 12% (n = 9) of affected patients.

As shown in Table 1, patients with DD were older and more likely to have hypertension than unaffected patients. Both echo and cine-CMR demonstrated that DD was accompanied by progressive systolic dysfunction as measured by LVEF or wall motion score (p = 0.001). Consistent with this, DE-CMR demonstrated a stepwise increase in infarct size in relation to graded severity of DD (p = 0.005). Whereas LV mass was similar between groups, there was a direct relationship between diastolic grade and left atrial size measured by either echo linear dimensions or cine-CMR volumes (p ≤ 0.005).

**Table 1:** Clinical and Conventional Imaging Characteristics

	Normal Diastolic Filling (n = 40)	Grade 1 Diastolic Dysfunction (n = 21)	Grade 2 Diastolic Dysfunction (n = 45)	Grade 3 Diastolic Dysfunction (n = 9)	P
**CLINICAL**					
**Age (year)**	50 ± 10	62 ± 7	61 ± 14	52 ± 12	**< 0.001**
**Male gender**	90% (36)	86% (18)	71% (32)	100% (9)	0.05*
**Atherosclerosis Risk Factors**					
Hypertension	28% (11)	43% (9)	62% (28)	22% (2)	**0.007**
Hyperlipidemia	33% (13)	38% (8)	60% (27)	56% (5)	0.06*
Diabetes Mellitus	18% (7)	10% (2)	27% (12)	22% (2)	0.41
Tobacco Use	40% (16)	24% (5)	40% (18)	33% (3)	0.58
Family History	38% (15)	24% (5)	18% (8)	22% (3)	0.22
**Coronary Artery Disease History**					
Prior Myocardial Infarction	3% (1)	14% (3)	4% (2)	0% (-)	0.20
Prior Coronary Revascularization	5% (2)	19% (4)	11% (5)	11% (1)	0.40
**Cardiovascular Medications**					
Beta-blocker	100% (40)	100% (21)	98% (44)	100% (9)	0.67
ACE Inhibitor/ARB	73% (29)	62% (13)	82% (37)	67% (6)	0.33
HMG-CoA Reductase Inhibitor	98% (39)	100% (21)	93% (42)	100% (9)	.47
Aspirin	100% (40)	100% (21)	100% (45)	100% (9)	-
Thienopyridines	98% (39)	95% (20)	93% (42)	100% (9)	0.72
**Infarct Related Artery**					
Left Anterior Descending	58% (23)	62% (13)	64% (29)	78% (7)	0.71
Right Coronary	33% (13)	24% (5)	29% (13)	11% (1)	0.60
Left Circumflex	10% (4)	14% (3)	7% (3)	0% (-)	0.57
**Revascularization Strategy**					
Primary Percutaneous Intervention	73% (29)	62% (13)	67% (30)	56% (5)	0.72
Primary Thrombolysis	25% (10)	43% (9)	27% (12)	44% (4)	0.36
**ECHOCARDIOGRAPHY**					
**LV Morphology/Systolic Function**					
Ejection Fraction (%)	50 ± 8	50 ± 11	48 ± 12	32 ± 15	**< 0.001**
Wall Motion Score	13 ± 10	14 ± 11	15 ± 12	33 ± 14	**< 0.001**
End-diastolic diameter (cm)	5.6 ± 0.5	5.8 ± 0.5	5.6 ± 0.5	6.2 ± 0.4	**0.01**
End-systolic diameter (cm)	4.1 ± 0.5	4.3 ± 0.6	4.2 ± 0.6	5.1 ± 0.7	**< 0.001**
Relative Wall Thickness (g/m^2^)	.030 ± 0.04	0.29 ± 0.03	0.31 ± 0.04	0.25 ± 0.04	**0.005**
Myocardial Mass (g/m^2^)	92 ± 15	97 ± 14	96 ± 15	92 ± 16	0.56
**Left Atrial Diameter **(cm)	3.8 ± 0.5	3.9 ± 0.6	4.0 ± 0.5	4.4 ± 0.4	**0.005**
**CARDIOVASCULAR MAGNETIC RESONANCE**					
**LV Morphology/Systolic Function**					
Ejection fraction (%)	57 ± 8	55 ± 9	54 ± 12	41 ± 10	**0.001**
Wall motion Score	11 ± 9	14 ± 7	15 ± 10	29 ± 12	**< 0.001**
End-diastolic volume (ml)	151 ± 34	141 ± 32	149 ± 45	220 ± 49	**< 0.001**
End-systolic volume (ml)	66 ± 24	66 ± 26	70 ± 36	133 ± 49	**< 0.001**
Myocardial Mass (g/m^2^)	67 ± 12	71 ± 14	72 ± 17	79 ± 20	0.15
**LV Infarct Size **(% myocardium)	14 ± 9	16 ± 9	17 ± 10	27 ± 8	**0.005**
**Left Atrial Volume **(ml)	80 ± 19	77 ± 20	95 ± 31	115 ± 25	**< 0.001**

Boldface type indicates p value < 0.05

* indicates p < 0.1

### Automated cine-CMR Segmentation

LV-METRIC successfully generated filling curves in all cases, yielding multiple diastolic parameters (Table 2). Figure 1 provides a representative example of automated cine-CMR segmentation by LV-METRIC (Figure 1A) as well as a resultant LV filling curve (Figure 1B). E and A waves, obtained by taking the first derivative of the volume-time curve, were discernable in 94% (n = 108) of patients. Figure 2 provides typical E:A filling profiles generated by LV-METRIC in relation to graded severity of echo-evidenced DD.

**Table 2:** Diastolic Filling Parameters

	Normal Diastolic Filling (n = 40)	Grade 1 Diastolic Dysfunction (n = 21)	Grade 2 Diastolic Dysfunction (n = 45)	Grade 3 Diastolic Dysfunction (n = 9)	P
**Cardiovascular Magnetic Resonance**					
E:A Ratio	3.1 ± 2.4	1.1 ± 0.4	1.9 ± 1.4	4.8 ± 3.3	**< 0.001**
Diastolic Volume Recovery (% diastole)	65 ± 16	81 ± 5	73 ± 13	58 ± 15	**< 0.001**
Time to Peak Filling Rate (msec)	174 ± 119	321 ± 205	221 ± 136	136 ± 36	**0.001**
Peak Filling Rate (ml/sec)	266 ± 76	207 ± 58	231 ± 73	353 ± 91	**< 0.001**
Normalized Peak Filling Rate	3.3 ± 1.2	2.7 ± 0.6	3.0 ± 0.7	4.1 ± 0.8	**0.001**

**Echocardiography**					
E:A Ratio	1.4 ± 0.4	0.9 ± 0.3	1.3 ± 0.3	3.1 ± 1.2	**< 0.001**
Deceleration time (msec)	187 ± 33	249 ± 41	185 ± 40	127 ± 16	**< 0.001**
Isovolumic relaxation time (msec)	90 ± 12	96 ± 15	89 ± 17	73 ± 11	**0.02**
Tissue Doppler e'/a' (septal)	1.1 ± 0.3	0.6 ± 0.2	0.8 ± 0.3	1.2 ± 0.5	**< 0.001**
Tissue Doppler e'/a' (lateral)	1.8 ± 0.6	0.8 ± 0.3	1.0 ± 0.5	1.9 ± 0.6	**< 0.001**
E/e' (mean e')	6.3 ± 2.0	7.8 ± 2.6	9.5 ± 2.6	15.0 ± 8.9	**< 0.001**

Repeat LV-METRIC processing was performed in 30 sequential patients to assess reproducibility (Table 3). All diastolic variables were highly reproducible, with ≤ 1% difference between readers for DVR_80_, TPFR, and PFR. Mean processing time was 2:09 ± 0:51 minutes for the initial reader (175 ± 34 images per exam), with similar processing time (2:15 ± 0:50) for the second reader (p = 0.12).

**Table 3:** Reproducibility of CMR Indices*

		*Intra-Observer Reproducibility*			*Inter-Observer Reproducibility*		
	**Initial**	**Repeat**	**Δ ± SD**	**p**	**Repeat**	**Δ ± SD**	**p**
**Diastolic Volume Recovery **(%)	69 ± 15	71 ± 15	1.5 ± 5.6	0.16	69 ± 16	0.2 ± 1.7	0.58
**Time to Peak Filling Rate **(msec)	231 ± 177	232 ± 176	1.3 ± 6.9	0.33	233 ± 179	1.5 ± 8.4	0.33
**Peak Filling Rate **(ml/sec)	243 ± 91	245 ± 94	2.2 ± 10.1	0.25	246 ± 92	2.8 ± 11.5	0.19
**E/A Ratio**	2.5 ± 1.9	2.5 ± 1.9	0.1 ± 0.7	0.52	2.9 ± 2.3	0.4 ± 1.5	0.17
**Processing Time **(minutes)	2:09 ± 0:51	2:06 ± 0:46	0:03 ± 0:17	0.28	2:15 ± 0:50	0:06 ± 0:21	0.12

* reproducibility and processing times assessed in subgroup of 30 consecutive patients

### Impact of Diastolic Dysfunction on Left Ventricular Filling

**Figure 3:**
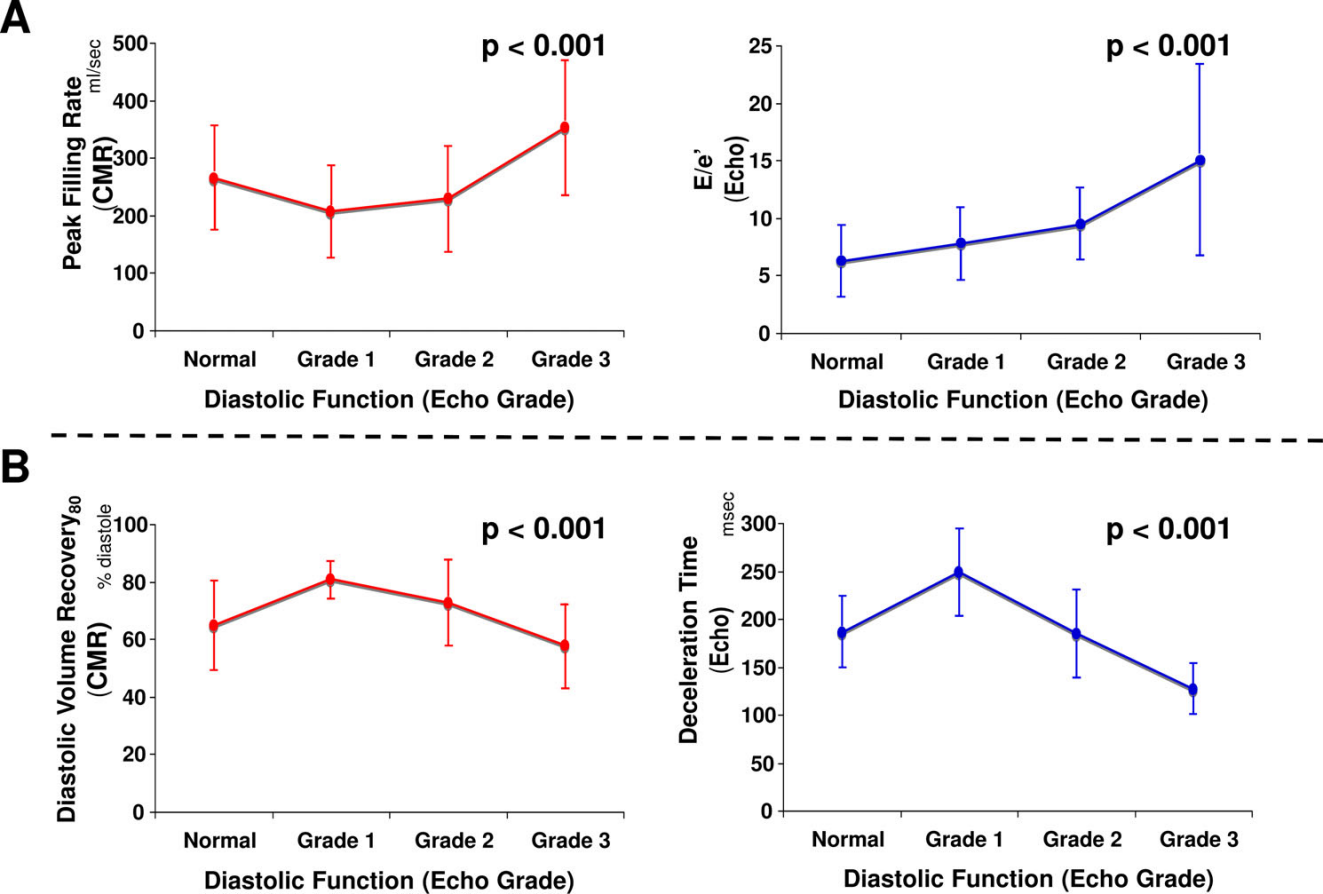
**Diastolic Filling Indices in Relation to Severity of Diastolic Dysfunction**. Diastolic indices by cine-CMR (red) and echo (blue) stratified by echo-assigned diastolic grade. **(A) **PFR by cine-CMR paralleled E/e' by echo. **(B) **DVR_80 _by cine-CMR paralleled echo-evidenced deceleration time (data presented as mean ± SD).

Table 2 reports CMR and echo diastolic parameters for patients stratified according to graded severity of DD, demonstrating similar patterns for the two modalities. Figure 3 provides a side-by-side comparison of echo and CMR-evidenced diastolic filling parameters (Figure 3A) and timing intervals (Figure 3B) stratified by graded DD severity. Whereas E/e' by echo and PFR by cine-CMR (Figure 3A) increased with transition from grade 1 through grade 3 DD, deceleration time and DVR_80 _(Figure 3B) manifested a biphasic relationship - increasing among patients with grade 1 and decreasing among patients with grade 2 and 3 DD. Among the overall population, DVR_80 _modestly correlated with echo-evidenced deceleration time (r = 0.25, p = 0.01).

### E:A Profiles

E:A filling ratios by CMR and echo (Table 2) also yielded similar patterns in relation to graded DD severity. For both modalities, ratios were diminished with grade 1, progressively increased with grade 2, and augmented with grade 3 DD. When classified by the standard of echo-assigned diastolic grade, E:A ratios by cine-CMR were lower among patients with grade 1 DD compared to those with normal diastolic filling (1.1 ± 0.4 vs. 3.1 ± 2.4, p < 0.001), reflecting a similar relationship for echo-derived E:A ratios between these groups (0.9 ± 0.3 vs. 1.4 ± 0.4, p < 0.001). Conversely, when patients with grade 3 DD were compared to normals, E:A ratios by cine-CMR tended to be higher among the DD group (4.8 ± 3.3 vs. 3.1 ± 2.4, p = 0.099), again paralleling echo-derived E:A ratios (3.1 ± 1.2 vs. 1.4 ± 0.4, p < 0.01).

Among patients with echo-evidenced grade 2 DD, cine-CMR demonstrated E:A ratios > 1 in 68% (28/41; n = 4 with non-discernable E:A profiles). For echo, magnitude of E:A ratios was similar between patients with grade 2 DD and normal diastolic filling (1.3 ± 0.3 vs. 1.4 ± 0.4, p = 0.16). For cine-CMR, E:A ratios were slightly lower among the DD group (1.9 ± 1.4 vs. 3.1 ± 2.4, p < 0.01), consistent with decreased volumetric filling during early diastole. As further evidence of impaired early diastolic filling, patients with grade 2 DD had longer DVR_80 _(73 ± 13 vs. 65 ± 16, p = 0.01) and lower PFR (231 ± 73 vs. 266 ± 76, p = 0.04) when compared to normals.

### Diastolic Performance of cine-CMR Indices

To evaluate diagnostic performance of CMR indices in relation to echo DD severity, parameters were tested among each discrete group of echo-assigned diastolic grade and the common group of patients with normal echo-evidenced diastolic filling. For DVR_80_, the previously established normative threshold of < 77% diastole (to recover 80% stroke volume) was applied based on our prior research among controls with normal systolic/diastolic function [15]. For all other CMR indices, diagnostic thresholds were determined using a cutoff necessary to achieve a matched specificity (83%) to DVR_80_.

Diagnostic performance of CMR indices varied according to graded severity of echo-evidenced DD. DVR_80 _yielded a sensitivity of 71% for patients with grade 1 but did not identify any patients with grade 3 DD (Table 4). Conversely, at a matched specificity of 83%, stroke volume adjusted nPFR yielded a sensitivity of 67% for grade 3 but did not identify any patients with grade 1 DD, with similar results for unadjusted PFR (Table 5). Whereas PFR or DVR_80 _alone identified fewer than half of patients with echo-evidenced grade 2 DD, use of the two parameters as aggregate criteria identified 53% (24/45) of affected patients.

**Table 4:** Performance of cine-CMR Indices in Relation to Diastolic Dysfunction Severity - Diastolic Filling Intervals

	Threshold	Sensitivity	Specificity	Accuracy	Positive Predictive Value	Negative Predictive Value
**GRADE 1 (mild)**						
Diastolic Volume Recovery_80_	77%*	71% (15/21)	83% (33/40)	79% (48/61)	68% (15/22)	85% (33/39)
Time to Peak Filling Rate	**221 msec†**	48% (10/21)	83% (33/40)	70% (43/61)	59% (10/17)	75% (33/44)
**GRADE 2 (moderate)**						
Diastolic Volume Recovery_80_	**77%**	47% (21/45)	83% (33/40)	64% (54/85)	75% (21/28)	58% (33/57)
Time to Peak Filling Rate	**221 msec**	29% (13/45)	83% (33/40)	54% (46/85)	65% (13/20)	51% (33/65)
**GRADE 3 (severe)**						
Diastolic Volume Recovery_80_	**77%**	0% (0/9)	83% (33/40)	67% (33/49)	0% (0/7)	79% (33/42)
Time to Peak Filling Rate	**221 msec**	0% (0/9)	83% (33/40)	67% (33/49)	0% (0/7)	79% (33/42)

**Table 5:** Performance of cine-CMR Indices in Relation to Diastolic Dysfunction Severity - Diastolic Filling Rates

	Threshold	Sensitivity	Specificity	Accuracy	Positive Predictive Value	Negative Predictive Value
**GRADE 1 (mild)**						
Peak Filling Rate	**344 ml/sec†**	5% (1/21)	83% (33/40)	56% (34/61)	13% (1/8)	62% (33/53)
Normalized Peak Filling Rate	**4.02/sec†**	0% (0/21)	83% (33/40)	54% (33/61)	0% (0/7)	61% (33/54)
**GRADE 2 (moderate)**						
Peak Filling Rate	**344 ml/sec**	7% (3/45)	83% (33/40)	42% (36/85)	30% (3/10)	44% (33/75)
Normalized Peak Filling Rate	**4.02/sec**	9% (4/45)	83% (33/40)	44% (37/85)	36% (4/11)	45% (33/74)
**GRADE 3 (severe)**						
Peak Filling Rate	**344 ml/sec**	56% (5/9)	83% (33/40)	78% (38/49)	42% (5/12)	89% (33/37)
Normalized Peak Filling Rate	**4.02/sec**	67% (6/9)	83% (33/40)	80% (39/49)	46% (6/13)	91% (33/36)

* Threshold of 77% diastole (to recover 80% stroke volume) based on previously established normative cutoff [15]

† Matched to achieve equivalent specificity vs. DVR_80_

Consistent with diagnostic performance results, multinomial logistic regression demonstrated that prolonged diastolic filling intervals and increased diastolic filling rates were associated with different aspects of echo-evidenced diastolic dysfunction. As shown in Table 6, prolonged DVR_80 _was associated with presence of grade 1 (OR 2.79 per 10 point increment, CI 1.65-4.05, p = 0.001) with a similar trend for grade 2 (OR 1.35, CI 0.98-1.74, p = 0.06), whereas high PFR was associated with grade 3 DD (OR 1.14 per 10 ml/sec, CI 1.02-1.25, p = 0.02).

**Table 6:** Functional CMR Predictors of Diastolic Dysfunction Severity

	Variable	Odds Ratio	95% Confidence Interval	P
**GRADE 1 (mild)**				
	Diastolic Volume Recovery80*	2.79	1.65 - 4.05	**0.001**
	Peak Filling Rate†	0.95	0.86 - 1.04	0.28
**GRADE 2 (moderate)**				
	Diastolic Volume Recovery80	1.35	0.98 - 1.74	0.06
	Peak Filling Rate	0.96	0.89 - 1.03	0.24
**GRADE 3 (severe)**				
	Diastolic Volume Recovery80	1.00	0.51 - 1.51	1.00
	Peak Filling Rate	1.14	1.02 - 1.25	**0.02**

Model χ^2 ^= 43.3, p < 0.001

* Per 10 point increment

† Per 10 ml/sec increment

## Discussion

This study demonstrates that automated processing of routine cine-CMR can discern physiologic changes that occur with graded severity of DD. LV-METRIC generated LV filling curves within an average processing time of 2 minutes and yielded highly reproducible indices. Diastolic filling ratios (E:A), timing intervals (DVR_80_), and filling rates (PFR) paralleled echo findings relative to DD grade. Diastolic filling (DVR_80_) was prolonged in patents with grade 1 and shortened with grade 3 DD, paralleling echo-evidenced DT. Diastolic filling rates (PFR, nPFR) increased with DD grade, similar to E/e' by echo. At matched specificities of 83%, DVR_80 _identified 71% of patients with grade 1 but no patients with grade 3 DD, while nPFR identified a similar proportion (67%) with grade 3 but no patient with grade 1 DD.

Diastolic indices generated by LV-METRIC were concordant with established concepts regarding filling changes that occur with graded DD severity [4,22,23]. For grade 1, impaired LV relaxation results in decreased early (E) filling, increased atrial (A) filling, and prolonged time from end-systole to peak diastolic filling. For grade 2 (pseudonormalization), the combination of impaired LV relaxation, decreased compliance, and increased LA pressure results in a seemingly normal E:A filling pattern. For grade 3 (restrictive filling), impaired LV relaxation and compliance are offset by a marked increase in LA pressure, producing an increased E:A ratio with an increased filling rate but shortened duration of early LV filling and a reduction in late diastolic filling. Our results were consistent with these physiologic concepts. Data generated by LV-METRIC demonstrated a decrease in CMR E:A ratio (1.1 ± 0.4) for patients with grade 1, and stepwise increase among patients with grade 2 (1.9 ± 1.4) and grade 3 (4.8 ± 3.3) (p < 0.001). Diastolic filling intervals (DVR_80_, TPFR) were prolonged in grade 1 and correlated with echo-evidenced DT. PFR was increased and TPFR decreased among patients with grade 3 DD, consistent with augmented early diastolic filling in restrictive physiology.

LV filling indices derived from invasive angiography and radionuclide imaging have been used to assess diastolic performance [7-10,24]. Consistent with our cine-CMR results, invasive studies have shown that PFR is decreased in impaired LV relaxation and increased with progressive LV stiffness and augmented LA-LV diastolic filling gradients. This concept was demonstrated by Ohno et al, who used a pacing-induced heart failure model to demonstrate that peak early diastolic filling rate initially decreases and subsequently increases with progressive heart failure [25]. In this study, peak filling rate correlated with increased LA-LV filling gradients and was inversely related to LV stiffness. PFR measured invasively has also been shown to correlate with diastolic atrio-ventricular filling gradients and LV relaxation rates [26]. Studies using radionuclide angiography have reported reduced PFR, increased TPFR, and prolonged early filling intervals among coronary artery disease patients with preserved systolic function, suggesting that these changes may reflect one aspect of DD [27,28].

Our results extend upon prior studies that have demonstrated feasibility of DD assessment by cine-CMR. Maceira et al reported age-associated decrements in PFR among a normative cohort [29]. However, as this study was restricted to asymptomatic subjects without cardiac disease, normal and abnormal diastolic indices were not compared. Other studies have reported differences in PFR and TPFR among patients grouped by binary presence or absence of echo-evidenced DD [15,30]. However, these studies did not examine relations between DD severity and cine-CMR indices. Our study, conducted in a broad post-MI cohort with variable severity of DD, demonstrates that different aspects of LV filling by cine-CMR can be used to distinguish between grades of echo-evidenced DD. We are unaware of any prior study that has assessed the relation between graded severity of DD and global LV filling by any non-invasive imaging modality.

While multiple techniques can be used to assess LV filling, there are several potential advantages to cine-CMR. First, unlike radionuclide imaging, CMR entails no ionizing radiation and is thereby well-suited for serial assessment of diastolic performance. Our results demonstrated that cine-CMR indices were highly reproducible, with ≤ 1% inter-reader differences for multiple parameters (DVR_80_, TPFR, PFR). Second, while LV-METRIC is a novel segmentation algorithm, the actual cine-CMR data required is a standard component of nearly all clinical CMR exams and requires no tailored pulse sequences or imaging hardware. This enables our approach to be widely applied to both prospective and pre-existing exams. Our prior study demonstrated feasibility among a retrospective dataset of patients with normal systolic function [15]. Our current study extends upon this by demonstrating that LV-METRIC can discern diastolic filling changes among a broad population with varying severity of systolic dysfunction and diastolic impairment. Third, unlike echo, CMR can directly assess myocardial tissue composition and thereby assess the influence of infarcted myocardium on diastolic performance. Our results demonstrated a stepwise increase in infarct size with graded severity of DD, suggesting that changes in myocyte tissue composition may alter LV compliance and contribute to DD.

Several limitations of our study should be recognized. In our study, LV volumes were assessed using short axis images acquired with 6 mm slice thickness and 4 mm gap. It is possible that gaps between LV slices could impact volumetric assessment. Future studies incorporating high-resolution 3D cine-CMR for volumetric assessment of LV diastolic function are planned to address this issue. Additionally, while our findings demonstrate that automated processing of routine cine-CMR can discern filling changes that occur with increasing severity of DD, approximately 1/3 of patients with grade 1 or grade 3, and nearly 1/2 of patents with grade 2 DD were not classified as abnormal by cine-CMR. This may be attributable to differences in temporal resolution between modalities and it is possible that diagnostic results would have been better if dedicated, higher temporal resolution cine-CMR were performed. However, prior studies have reported that LV filling indices generated by high resolution RNCA were discordant with echo in 16% of cases, suggesting that differences between modalities in our study are partially attributable to the approach used for diastolic assessment [31]. Our segmentation approach focused solely on LV filling, which may be limited if the LA compensates to preserve normal LV diastolic filling. This may be addressed through LA segmentation, which would be particularly useful to identify physiologic changes in grade 2 DD. As LV-METRIC utilizes routine cine-CMR data, future applications may combine LV and LA segmentation for integrated assessment of diastolic performance.

## Conclusions

In conclusion, this study demonstrates that LV filling curves generated by automated processing of routine cine-CMR can be used to discern volumetric filling changes that occur with graded severity of DD. Patients with grade 1-2 had prolonged filling intervals whereas patients with grade 3 dysfunction had increased diastolic filling rates. Future studies are needed to ascertain whether cine-CMR indices can be used to guide therapeutic management and stratify clinical outcomes for patients at risk for DD and related complications.

## Abbreviations

DD: diastolic dysfunction; CMR: cardiovascular magnetic resonance; PFR: peak filling rate (absolute); nPFR: normalized peak filling rate (stroke volume adjusted); TPFR: time to peak filling rate; DVR: diastolic volume recovery;

## Competing interests

The authors' institution has submitted a patent for the automated segmentation algorithm (LV-METRIC) described in this study.

## Authors' contributions

DDM participated in the study's design and coordination, image analysis, data collection, and manuscript preparation. NCFC and YW developed the automated CMR segmentation software (LV-METRIC) used for image analysis. SS, KK, SJM, JKM, TML, MRP and RBD contributed to image/data analysis and study design. JWW conceived of the study, participated in its design and coordination, and drafted the manuscript. All authors read and approved the final manuscript.
